# Annotation of glycoside hydrolases in unassembled metagenomes using CAZyO_GH_

**DOI:** 10.1093/bioadv/vbag137

**Published:** 2026-07-09

**Authors:** Nicholas G Griffin, Alison E Hughes, Daniel S Erdody, Eliott Berlemont, Shannon Sweeney, Tara Fareghbal, Klaus B Hagedorn, Renaud Berlemont

**Affiliations:** Department of Biological Sciences, California State University—Long Beach, Long Beach, CA, 90840-0004, United States; Department of Biological Sciences, California State University—Long Beach, Long Beach, CA, 90840-0004, United States; Department of Biological Sciences, California State University—Long Beach, Long Beach, CA, 90840-0004, United States; Department of Biological Sciences, California State University—Long Beach, Long Beach, CA, 90840-0004, United States; Department of Biological Sciences, California State University—Long Beach, Long Beach, CA, 90840-0004, United States; Department of Biological Sciences, California State University—Long Beach, Long Beach, CA, 90840-0004, United States; Department of Earth Sciences, California State University—Long Beach, Long Beach, CA, 90840-0004, United States; Department of Biological Sciences, California State University—Long Beach, Long Beach, CA, 90840-0004, United States

## Abstract

**Motivation:**

Functional characterization of microbiomes often relies on the sequencing of metagenomic DNA extracted from environmental samples, with current approaches using metagenome-assembled genomes (MAGs). Although glycoside hydrolases (GHs) are central to carbon cycling, accurate annotation of GHs in metagenomic datasets remains challenging due to the multidomain architecture of carbohydrate-active enzymes and the prevalence of unassembled short reads due to limitations in the MAG-generation process.

**Results:**

Here, we present CAZyO_GH_ (CAZymes Open-source GH annotation), a curated reference database for the domain-specific identification of 135 protein domains spanning 99 GH families with well-defined catalytic domain signatures. CAZyO_GH_ focuses on individual GH domains, enabling robust annotation of both assembled and unassembled metagenomic data. We validated CAZyO_GH_ by reanalyzing genomes listed in CAZy db, where predicted GH profiles closely matched reported values. Next, we used CAZyO_GH_ to analyze 12 human gut metagenomes and 12 newly sequenced soil microbiomes to reveal environment-specific GH repertoires. By accurately detecting catalytic domains independent of the genomic context, CAZyO_GH_ improves sensitivity and specificity in short-read metagenomic annotation. This framework provides a scalable and reproducible approach to investigate carbohydrate-active enzymes across ecosystems, advancing our capacity to characterize microbial functional potential in global carbon cycling.

**Availability and implementation:**

CAZyO_GH_ data is available on figshare (https://figshare.com/projects/CAZyO_GH/267770).

## 1 Introduction

Across environments, complex microbial communities (i.e. microbiomes) support essential biogeochemical cycles (e.g. carbon and nitrogen-cycling). More specifically, it is their genes and the enzymes they encode that carry out these processes. Among others, glycoside hydrolases (GHs) are carbohydrate active enzymes (CAZymes) essential for the processing of carbohydrates and thus support the nutrient cycling and the flux of energy across terrestrial and marine ecosystems and in the gut of mammals including humans, among others (Berlemont and Martiny 2016, Luo *et al.* 2020, Raza *et al.* 2023, Ge *et al.* 2025). Other CAZyme classes, including carbohydrate esterases (CEs) and polysaccharide lyases (PLs), also contribute to carbohydrate degradation. However, GHs represent the most diverse CAZyme class and frequently display complex multidomain architectures, making them particularly challenging to annotate in short-read metagenomic datasets.

Microbiome characterization often relies on the sequencing of metagenomic DNA directly extracted from environmental samples. As 2025, more often than not, the short-read sequences produced by high-throughput sequencing are assembled into contigs and then “metagenome assembled genomes” (MAGs) are combined and analyzed using standard genomic tools (e.g. gene calling and functional annotation) in order to investigate the functional potential (i.e. predicted function based on functional gene annotation) of individual lineages and of the microbial communities (Hyatt *et al.* 2010, Nayfach *et al.* 2015, Chivian *et al.* 2023). However, although contigs and MAGs reconstruction can reveal the genome structure and evolution of environmental lineages, they do not reflect the whole microbial communities. Indeed, some lineages can be difficult to assemble and simply not reported. For example, although *Streptomyces* (phylum Actinomycetota) is a well-known and abundant member of microbial communities in terrestrial ecosystems (e.g. Delgado-Baquerizo *et al.* 2018, Olanrewaju and Babalola 2019, Pombubpa *et al.* 2023), the “Global Earth Microbiomes” (GEM) lists only 3 medium quality *Stretpomyces*-related MAGs with an estimated degree of completeness ranging from ∼60 to 70%, and more than 600 contigs each, among the 52 123 newly created MAGs and although 750 of the initial metagenomes were derived from soil ecosystems (Nayfach *et al.* 2021).

Difficult to assemble environmental genomes from sequenced metagenomes result from the shallow sequencing of individual environmental genomes which reflect technical or financial limitations in DNA extraction and sequencing, complexity of microbial communities, and complexity of individual microbial genomes. More specifically, some samples contain low amount of DNA (Head *et al.* 2014, McNulty *et al.* 2020). While this limitation can be partially mitigated by isothermal multiple displacement DNA amplification combined with deep sequencing, this approach also has its limits. Notably, the increased cost of deep sequencing makes this method less suitable to analyze large quantities of samples, especially for research groups with limited funding (Hillmann *et al.* 2018). Next, the complexity of a microbial community also limits assemblies. Specifically, uneven microbial communities and individual strain variation within samples limit the construction of MAGs from low abundance lineages or organisms associated with complex and large genomes. Inflated sequence reads of abundant lineages often mask rare species and reduce the quality or prevent the creation of contigs and MAGs (Zhou *et al.* 2022). Furthermore, short-read assembly of diverse communities often result in fragmented outputs and mis-assembly, which makes MAG construction challenging (Yorki *et al.* 2023, Mirete *et al.* 2025). Finally, the complexity of individual genomes constitutes a major challenge for the creation of MAGs. For instance, species containing repetitive regions are difficult to assemble (Erkorkmaz *et al.* 2025) whereas large genomes (e.g. *Streptomyces*) can be challenging to assemble (Chu *et al.* 2013).

In contrast, unassembled (and unassemblable) short-read metagenomic DNA sequences contain valuable information about the entire microbial community. However, the processing of short-read dataset requires specialized databases to identify the microbial community composition such as Kaiju (Menzel *et al.* 2016) and Kraken (Wood *et al.* 2019), and to investigate their functional potential such as NCycDB (Tu *et al.* 2019) and PCycDB (Zeng *et al.* 2022), for the identification of traits involved in nitrogen and phosphorus-linked metabolism, respectively. Although general and GH-specific tools for short-read annotation have been developed in the past (e.g. Keegan *et al.* 2016, Berlemont *et al.* 2020), no stand-alone database is publicly available to identify GHs in short-read datasets despite the fact that these enzymes are essential across ecosystems.

**Figure 1:**
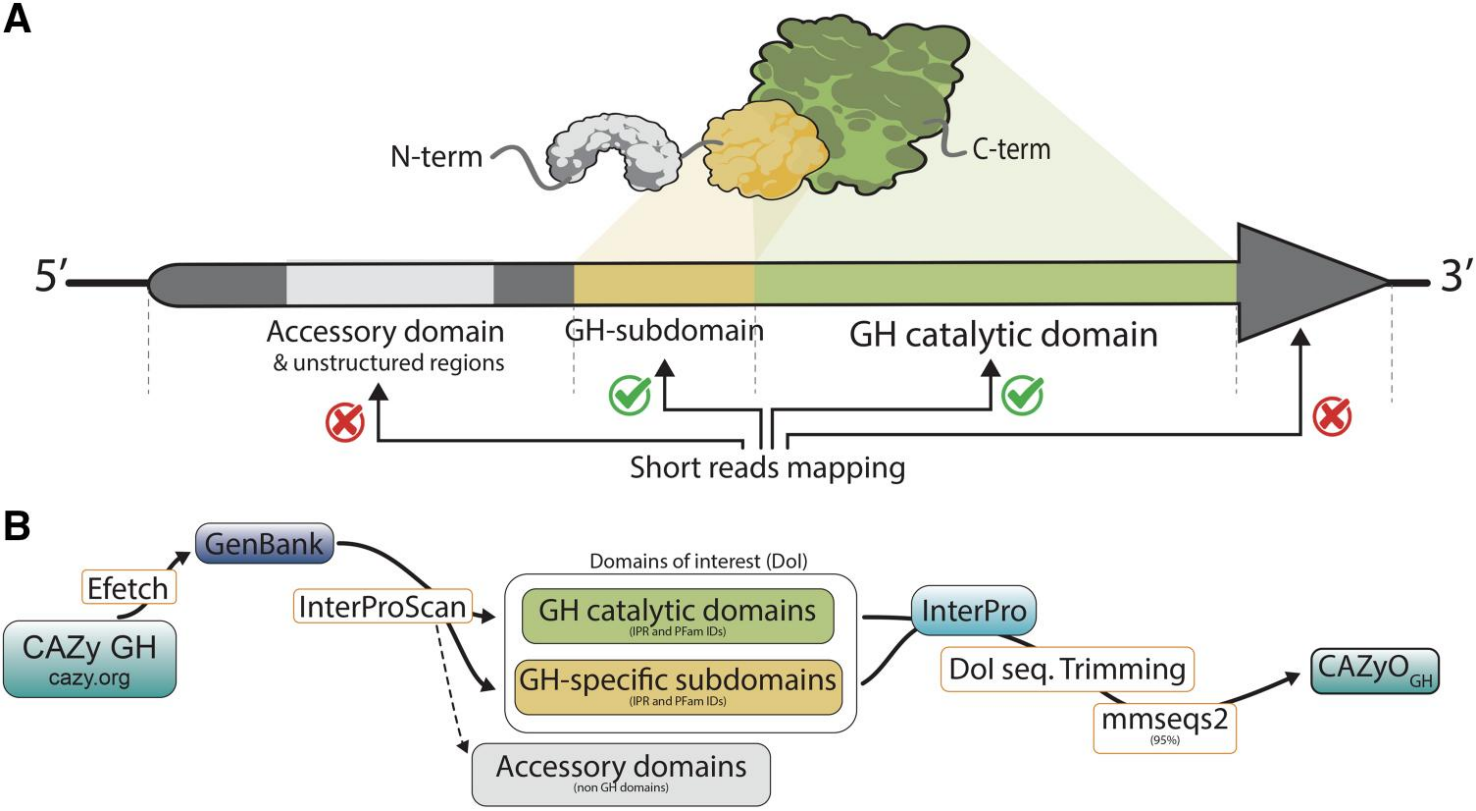
Overall CAZyO_GH_ mapping approach and database design. (A) Domain specific identification of GH according to CAZyO_GH_. (B) Construction pipeline of CAZyO_GH_.

The modular architecture of CAZymes, including the presence of catalytic and accessory domains, has been recognized for decades and forms the basis of CAZy classification (Henrissat and Davies 1997). However, this modularity presents challenges for sequence-based annotation (Nguyen *et al.* 2019), particularly in short-read metagenomic datasets where sequence context is limited. Briefly, many characterized CAZymes display accessory non-catalytic domains such as carbohydrate binding modules (CBMs) not endowed with enzymatic activity (Fig. 1A). In addition, because these accessory domains are distributed among multiple CAZyme families, identifying short-read sequences matching known proteins does not simply allow for the identification of GHs (or other specific domains of interest) (Fig. 1A). Consistent with this, the CAZy db (cazy.org) states that “[s]ome glycoside hydrolases are multifunctional enzymes that contain catalytic domains that belong to different GH families” (Drula *et al.* 2022). In addition, the complex architecture and variation in sequence length of multidomain GH proteins (Nguyen *et al.* 2019, Berlemont *et al.* 2022) can lead to mis-annotations since unassembled short reads lack the context necessary to make alignments and annotations confidently (Fraser *et al.* 2006). In this context, targeting catalytic domains directly, rather than full-length proteins, provides a more robust strategy for GH annotation in short-read datasets. However, a curated, domain-specific reference resource enabling this approach has been lacking.

Here, we developed CAZyO_GH_ database (CAZymes Open-source GH annotation), a curated reference annotation database for the domain-specific identification of 135 GH domains from 99 GH families (Fig. 1B). Although CAZyO_GH_ was designed for the precise annotation of GH catalytic domains in short-read unassembled metagenomes, it can also identify GH in assembled sequences (e.g. contigs, MAGs, and individual genomes). Briefly, CAZyO_GH_ was first validated by reannotating genomes listed on CAZy db. CAZyO_GH_-based functional annotation matched the data reported on CAZy db. Next, we used CAZyO_GH_ to re-annotate short reads and MAGs from 12 human gut metagenomes and from newly sequenced soil metagenomes. In these datasets, the CAZyO_GH_-based annotation highlighted (i) the broad GH repertoire in samples for which MAGs were available, (ii) the GHs distribution in new unassemblable soil metagenomes, and (iii) the uneven distribution of GHs across ecosystem types.

## 2 Methods

### 2.1 Data retrieval and curation

The protein sequences from GH listed on CAZy database (CAZy.org, June 2024) were retrieved from GenBank using Efetch tools (Sayers 2022) and reanalyzed with InterProScan (Jones *et al.* 2014) (Fig. 1B). Then proteins from each GH family were associated with a set of IPR (and PFam) domains (Table S1). The domain list was manually curated and a single IPR (and PFam) domain was associated with the GH catalytic domain or domain of interest (DoI, Table S1). Eventually, GH-specific non-catalytic subdomains were identified for some GH families. For example, a “C-terminal domain” (IPR041036/PF18564) was found only associated with some the GH5 catalytic domain (GH5, IPR001547, PF00150) albeit not systematically. Finally, few GH families described on CAZy db, had no unique IPR nor PFam ID and thus were not included. In total, 135 GH-specific domains were identified (Table S1). As a result, CAZyO_GH_ currently covers a subset of GH families for which domain definitions are publicly available and sufficiently resolved to support unambiguous identification.

Next, for each entry, we retrieved the complete set of sequences from InterPro and trimmed the sequence to retain the specified DoI only. Finally, the sequences for the DoI were clustered using mmseqs2 (version 15-6f452+ds-2) at a 95% similarly (Steinegger and Söding 2017) using the following options: “mmseqs cluster—cov-mode 0-c 0.95—min-seq-id 0.95 input.txt output.txt tmp” and representative sequences were combined in a single FASTA file.

### 2.2 CAZyO_GH_ validation

To evaluate CAZyO_GH_, we used the “genomes” section CAZy db as a reference (Drula *et al.* 2022). Specifically, a random set of 10 000 completely sequenced bacterial genomes cross-listed on CAZy db and the BV-BRC database (Olson *et al.* 2023) was identified and the corresponding assembled DNA sequences were retrieved from BV-BRC. Next, for each sequence file, we simulated short-read sequences using the ART Illumnia tool (Q version 2.5.8), specifying the MiSeqv3 tool, a coverage of 10×, and a short-read length of 200 base pairs (bp) using the following command: “art_illumina—sam-i input.fna-l 200-f 10—ss MSv3-o output” (Huang *et al.* 2012). Finally, the simulated short reads were BLASTed against CAZyO_GH_ using DIAMOND BLAST (Buchfink *et al.* 2021) and specifying an e-value cutoff of 10^−5^ with the following command: “diamond blastx-d CAZyO_GH-q input.fasta-o alignment_report.txt—evalue 1e-5.” A hit was deemed positive only if the top five best matches (based on e-value) were for the same domain family (i.e. GH catalytic domain or GH-specific subdomain). This consensus-based filtering step was designed to reduce false positives arising from ambiguous matches or shared domains across CAZyme families, increasing the specificity of domain assignments. Finally, the resulting hit distribution from each simulated short-read dataset was compared to the reported value listed on CAZy db.

### 2.3 Metagenomic datasets

First, 12 random human gut metagenomes datasets (i.e. Gut 1–12) were retrieved from the NIH-sequence read archive (SRA): SRR1930149 (Gut1), SRR4444828 (Gut2), SRR4783448 (Gut3), SRR5057053 (Gut4), ERR525699 (Gut5), ERR911987 (Gut6), ERR1190925 (Gut7), SRR4783607 (Gut8), ERR525746 (Gut9), ERR1190866 (Gut10), SRR5056792 (Gut11), SRR5056980 (Gut12). These datasets have been assembled as part of the genomic catalog of Earth’s microbiomes (Nayfach *et al.* 2021). Specifically, they correspond to 147 publicly available high (*n* = 51) and medium (*n* = 96) quality MAGs that we retrieved from the IMG/M portal at the Joint Genome Institute (https://portal.nersc.gov/GEM/).

Next, 12 original independent bulk soil samples from the Descanso Canyon, North of Avalon Bay on Santa Catalina Island—California were collected and the DNA sequenced. Briefly, three samples from the ridge (R, 33°21′0″ N, 118°20′3″ W) and 3 from the bottom (B, 33°20′58″ N, 118°20′4″ W) of the canyon were collected on August 10, 2022 (Su22) and February 15, 2023 (Wi23). All the samples were processed as follows: ∼50 gr of soil were collected aseptically 5–15 cm bellow the soil surface and kept in dry ice until being processed. Back in the Microbial Genomics Lab at CSU Long Beach—California, samples were incubated at room temperature for 30 min before processing. First, samples were sieved (1.981 mm sieve) to remove large debris (e.g. roots, twigs), homogenized using aseptically cleaned blender, and 0.25–0.28 gr were used for DNA extraction using the Qiagen DNAeasy PowerSoil extraction kit. Next, DNA extracts were processed at the UCI Genomics Research and Technology Hub (https://research.uci.edu/shared-facility/genomics-research-and-technology-hub/). DNA concentrations were normalized and metagenomic DNA libraries were created using Illumina DNA Prep kit and indexed with unique sets of dual barcodes. Finally, Paired-End sequencing (150 cycles) was performed on an Illumina NovaSeq 6000. The resulting FASTQ files containing 277 million reads (pre-QC) were uploaded to the SRA under the following accession numbers: SRR35620300–SRR35620311.

Finally, files from soil metagenomes were processed on KBase (Arkin *et al.* 2018, Chivian *et al.* 2023) for quality control (FastQC, v.1.2.2 with default parameters), taxonomic identification (Kaiju, v1.9.0 with default parameters), and in order to produce MAGs following an approach similar to the described in the genomic catalog of Earth’s microbiomes (Nayfach *et al.* 2021).

### 2.4 Short-read annotation and normalization

Metagenomic short reads (post-QC) were BLASTed against CAZyO_GH_ as described above to identify GH domains. In addition, to account for variation in domain length, hit counts were normalized as follows. First, we computed the average length of each domain of interest in CAZyO_GH_ (Table S1). Next, the domain lengths were compared to the domain length of GH13 (IPR006047/PF00128, 339 amino acids, L_GH13_), a large and ubiquitous GH domain. Specifically, domains were assigned a GH13-equivalent conversion factor (i.e. CF_x_=L_GH13_: L_GHx_) and individual raw hit counts (R_GHx_) were converted to normalized, GH13-equivalent, hit counts (N_GHx_ = R_GHx_ × CF_x_).

### 2.5 MAG annotation

In addition, to annotating the short reads, the retrieved MAG sequences were analyzed using two approaches. First, predicted protein sequences were analyzed using GeneHunt (Nguyen *et al.* 2019, Berlemont *et al.* 2022) and HMM profiles from Pfam V 37.4 (Finn *et al.* 2014) to identify the precise multidomain architecture of proteins with the domains of interest (i.e. GH). Next, predicted protein sequences were BLASTed against CAZyO_GH_ using DIAMOND BLASTp.

### 2.6 Statistics and data visualization

Data were processed and visualized using the R statistical programming interface (v. 4.3.3) and the following packages: reshape2 (v. 1.4.4), dplyr (v. 1.1.4), plyr (v. 1.8.9), splitstackshape (v. 1.4.8), ggplot2 (v. 3.5.2), forcats (v. 1.0.0), and stringr (v. 1.5.1).

## 3 Results

### 3.1 Database construction

CAZyO_GH_ was designed for the precise annotation of GH domains (i.e. domain of interest—DoI) rather than complete GH proteins (Fig. 1). As of Spring 2025, CAZyO_GH_ contains 1 615 517 trimmed, representative sequences for the 135 GH domain of interest accounting for 99 GH families. These families correspond to those with well-defined and unique domain signatures in InterPro/Pfam, enabling reliable domain-centric annotation. The number of sequences pertaining to each family varied, with well-described families accumulating upwards of over 100 000 sequences (e.g. GH13) whereas families defined recently contained reduced number of representative sequences (e.g. GH98). Domain lengths also varied extensively and ranged from 51 to 944 amino acids for GH112 (IPR035356/PF17386) and GH123 (IPR045711/PF19543), respectively. All sequences were compiled into a single FASTA file to be used as an open reference annotation database.

### 3.2 CAZyO_GH_ validation against CAZy db

**Figure 2:**
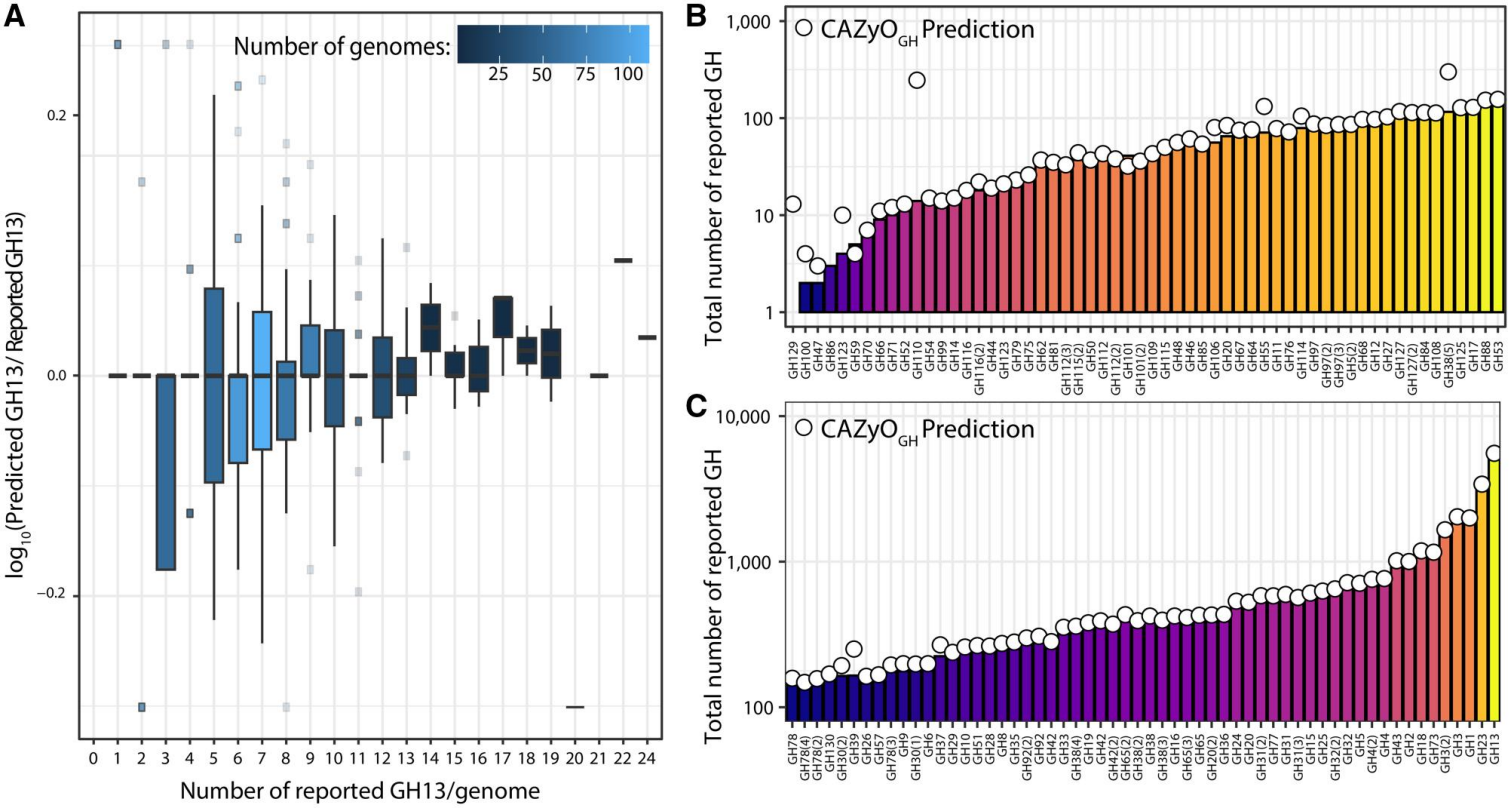
Comparison between reported and predicted GH domains in sequenced bacterial genomes. (A) Number of predicted (per CAZyO_GH_) vs. reported (per the CAZy db) GH13 in 1000 reanalyzed bacterial genomes. (B and C) Overall comparison of the total number of predicted (open circle) and reported (bar plot) GH domains in 1000 random reanalyzed bacterial genomes.

To evaluate CAZyO_GH_, we simulated 200 bp short-read DNA sequences from 10 000 randomly selected bacterial genomes listed on CAZy db and proceeded to their reannotation. Positive hits matching GH domains were defined as short reads for which at least the five best hits, with an E-value ≤10^−5^, matched the same domain family in CAZyO_GH_. Then, the number of raw hits, per simulated genome, was compared with the reported values from CAZy db. CAZyO_GH_-based identifications were systematically correlated with the reported value (Table S1) except for few GH families with scarce distribution in the analyzed genomes (e.g. GH7) or GH families created recently and associated with an overall reduced number of identified sequences (e.g. GH98) (Table S1). Next, focusing only on the GH domains that showed significant correlation with the CAZy-reported values, we performed a linear regression to link the raw GH hit counts per genome with the corresponding number of GHs reported in CAZy db (Table S1). Finally, another set of 1000 genomes, not part of the initial training dataset, was retrieved and processed as before. Then the raw hit counts were converted to actual number of GH genes per genome using the computed linear models and the number of predicted (using CAZyO_GH_) and reported (per the CAZy db) GHs were compared (Fig. 2).

When focusing on GH13 (Fig. 2A), the predicted and reported number matched across the board. However, some variations were observed, especially for the few genomes with extreme (>15) number of reported GH13. For these genomes, CAZyO_GH_ predicted more hits than reported. Detailed investigation suggested that in these genomes, the high number of GH13 results from gene duplication and recombination leading to multiple GH13 proteins eventually containing repeated GH13-segments (data not shown). Overall, when comparing the total number of predicted and reported GHs in the 1000 reanalyzed genomes, the number of predicted GHs matched or exceeded the number of reported GH in CAZy db (Fig. 2B and C).

Because domain annotation in metagenomic data does not readily lend itself to binary classification with well-defined true negatives, we evaluated performance based on the recovery of GH profiles relative to curated CAZy annotations across genomes. The strong agreement observed across both training and independent test datasets supports the robustness of the approach.

Across the board, CAZyO_GH_ predictions matched values reported by CAZy db, validating its use in further applications.

### 3.3 Human gut metagenomes reannotation

Short-read sequences, as well as MAGs, from 12 human gut microbiomes were retrieved and analyzed using CAZyO_GH_. First, although few GH domains were not identified, most domains of interest were detected albeit with variable frequency. This suggested that although GH are essential for the ecosystem functioning, not all the GHs are found in all the environments.

Next, we focused on GH families associated with multiple subunits (e.g. GH31, GH78). Overall, their sub-domain distributions strongly correlated (p_Spearman_<0.05) across samples. For example, per the InterPro database, most known GH30 proteins contain two conserved subdomains and eventually some variable accessory domains. The first GH30 core subdomain (IPR033453/PF02055) is 342 amino acids long on average, per CAZyO_GH_, whereas the second core subdomain (IPR033452/PF17189) is only 68 amino acids (Table S1). As, per InterPro database, these subdomains are almost systematically associated, the hit rates for the two core GH30 subdomains matched the expected ratio of ∼5:1 across samples (data not shown). The same trend was identified for other GH families, although with variable ratio. Yet, in few GH families (e.g. GH20, GH38, and GH81), the frequency of subdomains did not correlate across samples. Investigating the known multidomain architecture of these proteins using InterPro revealed that their subdomain composition was highly variable and that the corresponding subdomains were not systematically associated in these GH families.

Overall, to account for the variable length of the domain and subdomain of interest and the associated variable raw hit counts, we used GH13 (PF00128/IPR006047, 339 amino acids) an abundant and ubiquitous α-amylase, to normalize all the hits counts. The normalized, GH13-equivalent, hit counts provides a direct estimate of the GH-domain distribution.

**Figure 3:**
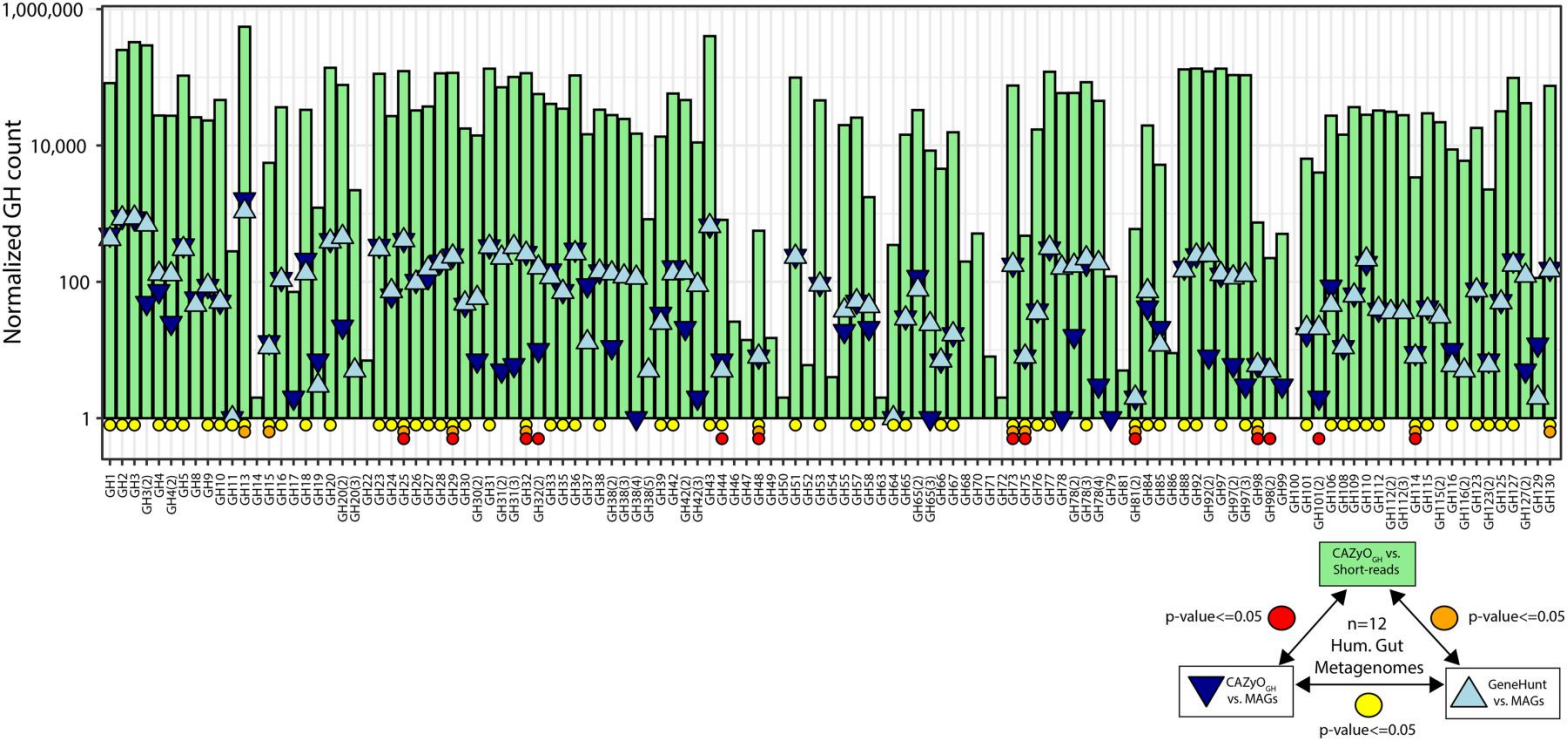
GH domains identification in 12 publicly accessible datasets derived from human gut. Normalized cumulative GH domain identification according to CAZyO_GH_ and GeneHunt.

Next, we compared HMM-based (GeneHunt) and BLAST-based (CAZyO_GH_) sequence identification of GH domain in the MAGs from the human gut microbiomes (Fig. 3). For most GH domains, GeneHunt identified more hits than CAZyO_GH_. However, across the board, the number of hits retrieved using GeneHunt correlated with the number of hits using CAZyO_GH_ (Fig. 3).

Finally, we compared the hits distributions between short-read annotation, using CAZyO_GH_, and MAGs annotations, across samples (*n* = 12). We identified 119 out of 135 domains of interest distributed across the 12 samples. As expected, the annotation of short reads identified orders of magnitude more hits (∼500× on average) than MAGs-based investigation. The identification of 103 domains, including the most abundant GH, in the short reads were not correlated with the identification of the corresponding domains in the MAGs. Overall, this indicates that MAGs annotation and short reads annotation using CAZyO_GH_ capture distinct and only partially overlapping components of the GH repertoire. This divergence is expected, as MAG-based approaches preferentially capture genes from abundant and well-assembled genomes, whereas short-read annotation retains signals from low-abundance or assembly-resistant taxa.

### 3.4 Soil metagenomes annotation

We sequenced 12 original soil microbiomes to further evaluate CAZyO_GH_ potential to investigate the functional potential in complex microbial communities. First, we uploaded the short reads on KBase for preprocessing (i.e. quality control, taxonomic profiling, and assembly) to mirror the approach employed in the catalog for Earth microbiome and in the analyzed human gut microbiome samples presented above. Although few contigs (<5 kbp) were generated, no MAG could be assembled. Thus, we focused on short reads for taxonomic and functional annotation.

**Figure 4:**
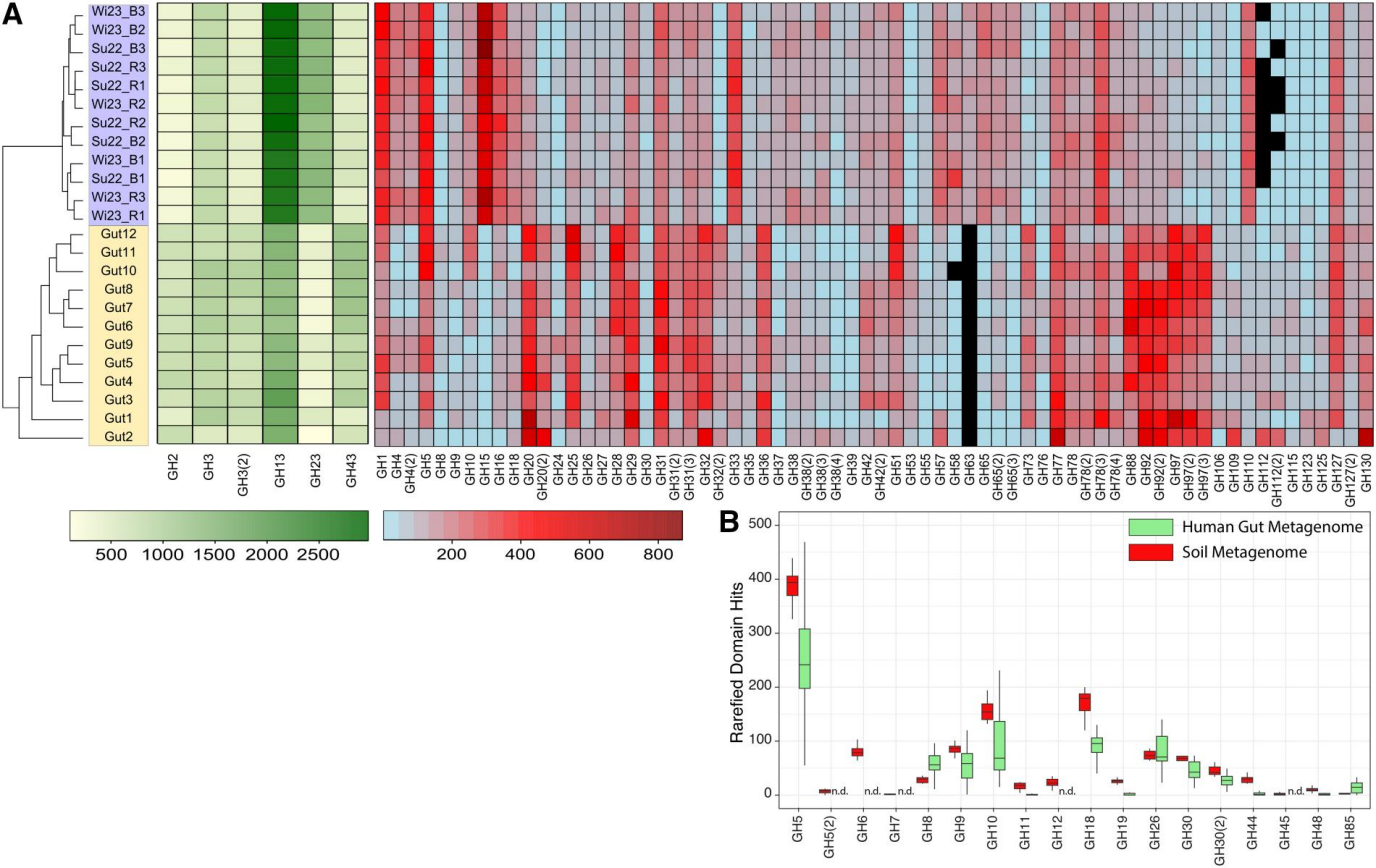
GH domains distribution in short read metagenomes. (A) Rarefied GH profiles across short reads from soil (*n* = 12) and human gut (*n* = 12) metagenomes. Highly abundant (yellow to green) and intermediate (blue to red) GH are displayed along with the sample clustering based on all the identified GH. The heatmap showing the distribution of the low abundance traits is available in supplementary data (Fig. S2). GH domains were categorized into high- (>400), intermediate- (>40), and low-abundance groups based on their mean normalized abundance across all samples (soil and gut combined). Because individual GH domains exhibit substantial variation across samples and ecosystems, the resulting categories are approximate and may partially overlap. (B) Hit counts of major GH families targeting cellulose, xylan, and chitin across metagenomes.

First, taxonomic profiling using Kaiju, implemented in KBase, revealed that the samples contained a complex microbial community systematically dominated by *Bradyrhizobium*, *Nocardioides*, and *Streptomyces*, among others. After rarefaction, the clustering of the samples highlighted the combined impact of the sample location (i.e. ridge or bottom of the canyon) and the season (i.e. Winter vs. Summer) (Fig. S1). Next, we used CAZyO_GH_ for the functional annotation of the QC’ed short reads as described above. As noted for the human gut metagenomes, although few domains were not detected, most GHs, including mostly cellulolytic GH6 and GH7, were identified in the soil samples albeit with highly variable frequencies (Fig. 4 and Fig. S2). These patterns suggest that soil microbiomes harbor a broader repertoire of GH families associated with the degradation of complex structural polysaccharides, consistent with the heterogeneous plant-derived carbohydrates present in terrestrial environments. In contrast, the more restricted GH profiles observed in gut metagenomes likely reflect the dominance of host-associated and diet-derived carbohydrates, which are typically more labile and compositionally constrained.

### 3.5 Ecosystem profiling

Next, CAZyO_GH_-based annotation of the short reads from the human gut and the soil metagenomes were compared (Fig. 4). First, samples from each environment clustered together thus suggesting ecosystem-specific GH-repertoires. Next, although very distinct, both the human gut and the soil samples were enriched in GH2, GH3, GH13, GH23, and GH43. The enzymes are frequently associated with oligosaccharides (GH2 and GH3), starch (GH13), peptidoglycan (GH23) and arabinoxylan (GH43) processing. Next, most GH with intermediate abundance showed uneven distributions in the human gut and in the soil suggesting that GH repertoires reflect ecosystem-specific carbohydrate inputs and microbial community composition (Fig. 4A). Specifically, the higher abundance of GH13 domains in soil metagenomes may reflect the presence of diverse α-glucans, including plant-derived starch residues, storage polysaccharides, and microbial glycogen, rather than direct evidence of higher starch utilization compared to the gut.

When focusing on GH families targeting structural polysaccharides such as cellulose, xylan, and chitin. More hits, from more GH families, were identified in soil than in the human gut microbiomes (Fig. 4B). In addition, specific domains including GH5(2), GH6, GH7, GH12, and GH45, were only identified in the soil metagenomes (Fig. S2). The presence of GH families targeting structural polysaccharides in both soil and gut microbiomes likely reflects fundamental processes of microbial biomass turnover, including the recycling of cell wall components such as peptidoglycan and chitin-like substrates, which are common across ecosystems. Together, these patterns suggest that GH profiles reflect a combination of ecosystem-specific inputs and conserved microbial metabolic functions.

## 4 Conclusion

CAZyO_GH_ provides a domain-centric framework for the precise annotation of glycoside hydrolases (GHs) across unassembled and assembled metagenomes. By focusing on catalytic domains rather than full-length, multi-domain CAZymes, CAZyO_GH_ minimizes misannotations arising from variable accessory modules and truncated short-read alignments. This approach enables accurate estimation of GH diversity and abundance directly from short-read datasets, allowing more comprehensive functional profiling of microbial communities across environments.

Across environments, CAZyO_GH_ revealed that the distribution of GH domains is highly uneven between ecosystems. In particular, human gut metagenomes were enriched in GH families linked to oligosaccharide and peptidoglycan degradation, whereas soil microbiomes displayed a broader and more diverse GH repertoire. Several domains, including GH6—typically associated with *Streptomyces* species (Book *et al.* 2016)—and GH7, which is characteristic of fungal cellulases (Hobdey *et al.* 2016), were detected almost exclusively in soil samples. These enzymes represent molecular signatures of soil ecosystems, reflecting the adaptation of microbial communities to the degradation of complex plant-derived polysaccharides and highlighting the ecological specificity of GH functional traits across environments. These patterns illustrate how CAZyO_GH_ enables the detection of ecologically meaningful functional signatures directly from short-read data, even in the absence of genome reconstruction.

CAZyO_GH_ emphasizes domain specificity where dbCAN (Zheng *et al.* 2023) and GeneHunt (Nguyen *et al.* 2019) rely primarily on HMM-based annotation of complete protein sequences or predicted ORFs, which can be biased toward assembled genomes (Yang *et al.* 2021) and overlook domain fragments common in unassembled metagenomes (Berlemont *et al.* 2020). In contrast, CAZyO_GH_ can detect GH signatures even when genome assembly is not feasible, capturing enzymes from low-abundance or from complex taxa that are often missing in MAG-based analyses. Consequently, CAZyO_GH_ complements dbCAN and GeneHunt by extending annotation capabilities to metagenomes where assembly is incomplete or impossible.

Nevertheless, several caveats remain. While domain-level annotation improves specificity, it cannot resolve the complete enzymatic context or substrate range of multi-domain CAZymes. Notably, the lack of correlation between MAG-based and short-read-based GH annotation does not reflect methodological inconsistency but rather fundamental differences in the underlying data structures. MAG reconstruction is biased toward abundant and easily assembled genomes, whereas short-read annotation captures genetic signals from low-abundance, highly fragmented, or assembly-resistant organisms. As a result, these approaches interrogate distinct fractions of the microbial community. Thus, rather than being contradictory, these approaches are complementary, and their combined use provides a more comprehensive view of microbial functional potential.

In addition, normalization by domain length partially mitigates length bias but may not fully account for read-depth variation across samples. Finally, while CAZyO_GH_ relies on manually curated InterPro–Pfam associations, it also addresses a broader limitation in the field: the annotation pipeline provided by CAZy db is not publicly accessible, and there is currently no direct or automated way to link CAZy data to public sequence repositories. Although CAZy db defines and distributes family-level information through its website (https://www.cazy.org/), the lack of transparent and programmatic integration restricts reproducibility and independent validation, challenges that CAZyO_GH_ helps to mitigate by offering an open, reference-based framework. In addition, GH families lacking unique or stable domain identifiers were not included, as their annotation at the domain level would be inherently ambiguous. However, as domain models are refined and expanded in public databases, CAZyO_GH_ can be extended to incorporate additional families. While CAZyO_GH_ currently focuses on GHs, the same domain-centric framework could be extended to other CAZyme classes, such as CEs and PLs, as domain definitions become sufficiently resolved for robust annotation.

Despite these limitations, CAZyO_GH_ represents a significant advance for GH annotation in environmental genomics. It facilitates high-resolution comparisons of carbohydrate-active enzyme repertoires across diverse microbiomes, from human gut to soil ecosystems. By bridging the gap between short-read metagenomic data and functional enzyme annotation, CAZyO_GH_ offers a scalable and transparent platform for future investigations into microbial carbon cycling and ecosystem metabolism.

## Supplementary Material

vbag137_Supplementary_Data

## Data Availability

The data underlying this article are available in figshare at (https://figshare.com/projects/CAZyO_GH/267770).
