# Supplementary material for: Annotation of glycoside hydrolases in unassembled metagenomes using CAZyOGH
Source: Bioinform Adv. 2026 Jul 9;6(1):vbag137. doi: 10.1093/bioadv/vbag137 (PMC13355591; doi:10.1093/bioadv/vbag137)
Supplement: vbag137_Supplementary_Data [file vbag137_supplementary_data.docx]

**
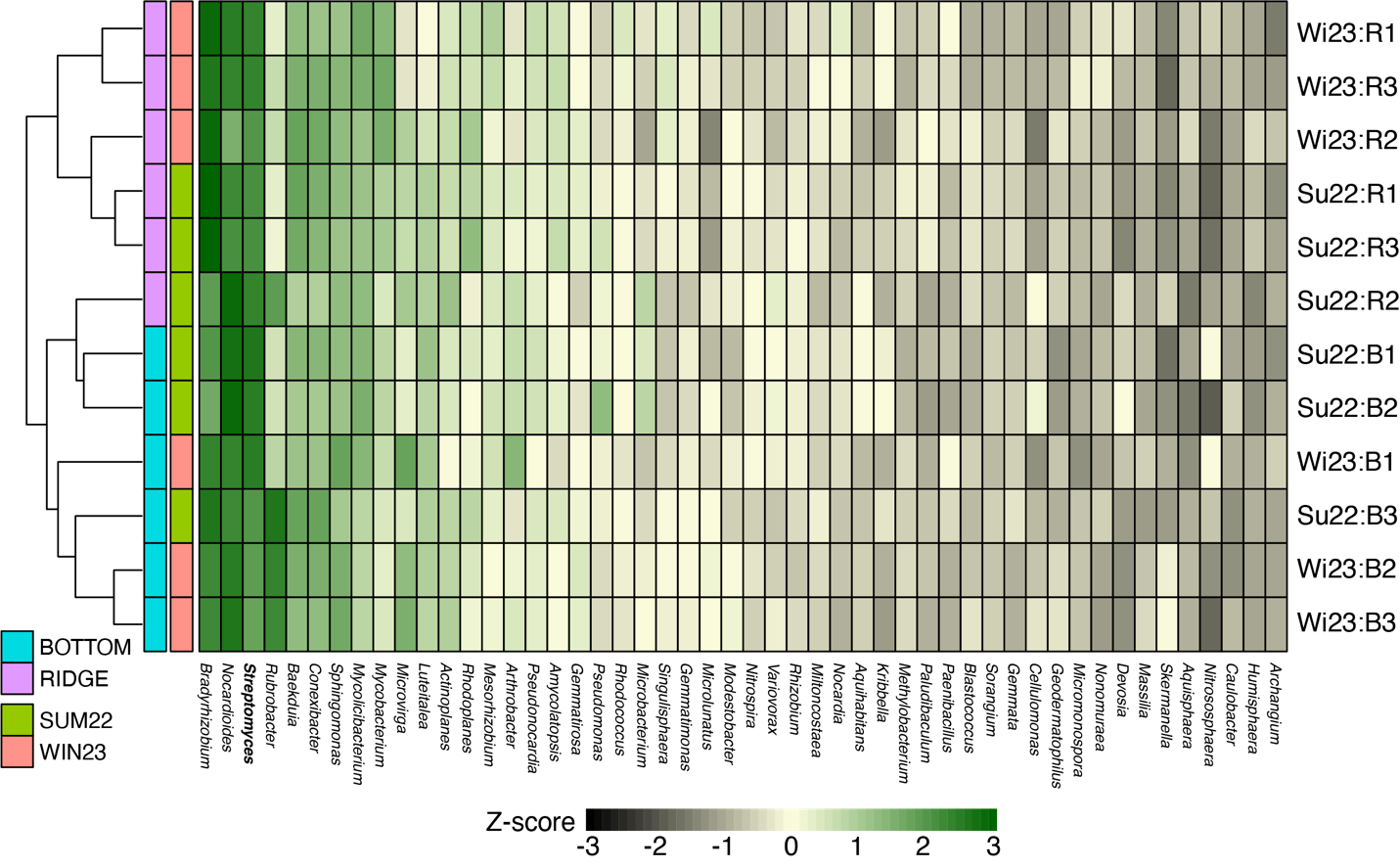
**

**Figure S1:** Distribution of major bacterial genera identified using Kaiju in the Descanso canyon (Santa Catalina Island, California, USA) soil metagenomes, after rarefaction (Su22: Summer 2022, Wi23: Winter 2023, R1-3: ridge of the canyon, B1-3: bottom of the canyon).

**
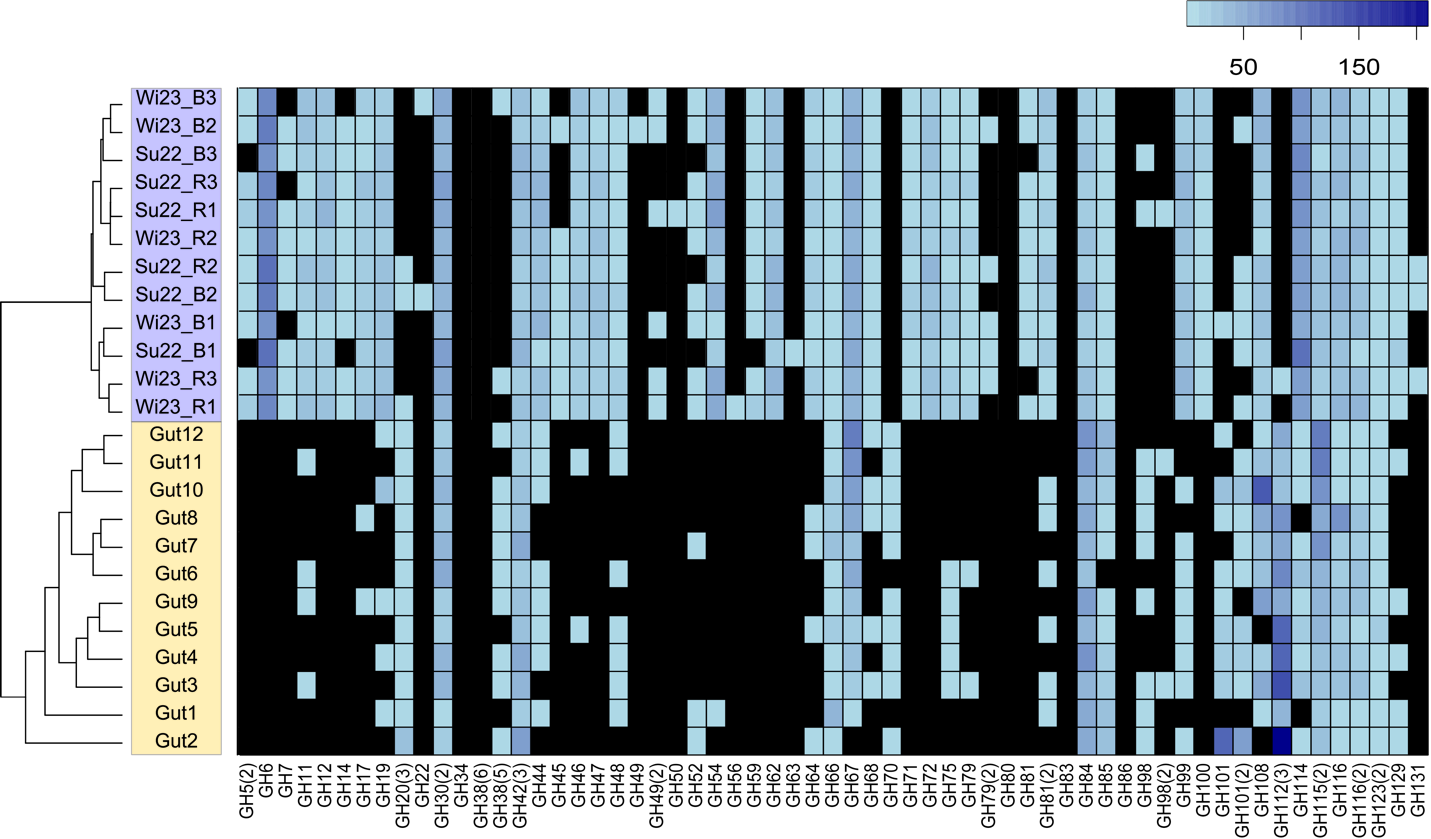
**

**Figure S2:** Low abundance GH domains distribution in short read metagenomes along with the sample clustering based on all the identified GH domains. Rarefied GH profiles across short reads from soil (n=12) and human gut (n=12) metagenomes. (Highly abundant and intermediate GH domains are displayed in Figure 4).

**Table S1.** GH domains on interest included in CAZyO_GH_, their ID number in the InterPro (IPR) and Pfam (PF) database (if available), average length (in amino acids) in the CAZyO_GH_ db, and the corresponding conversion factor (see main text). Correlation and linear regression statistics for the validation of CAZyO_GH_ (see main text).

| Domain | IPR/Pfam ID | Avg.  Length (AA) | Conv. Factor | Correlation | | Linear regression | | | |
| --- | --- | --- | --- | --- | --- | --- | --- | --- | --- |
|  |  |  |  | r_Pearson_ | p_Pearson_ | intercept | slope | r^2^ | p_regression_ |
| GH1 | IPR001360/PF00232 | 481 | 0.70 | 0.964 | <0.001 | 2.684 | 71.496 | 0.929 | 0.011 |
| GH2 | IPR006102/PF00703 | 104 | 3.27 | 0.788 | <0.001 | 9.342 | 11.482 | 0.621 | <0.001 |
| GH3 | IPR001764/PF00933 | 306 | 1.11 | 0.960 | <0.001 | 6.419 | 44.769 | 0.922 | <0.001 |
| GH3(2) | IPR002772/PF01915 | 262 | 1.29 | 0.916 | <0.001 | -18.881 | 35.250 | 0.839 | <0.001 |
| GH4 | IPR001088/PF02056 | 177 | 1.91 | 0.921 | <0.001 | 5.911 | 26.875 | 0.848 | <0.001 |
| GH4(2) | IPR022616/PF11975 | 218 | 1.55 | 0.921 | <0.001 | 7.502 | 31.335 | 0.849 | <0.001 |
| GH5 | IPR001547/PF00150 | 275 | 1.23 | 0.923 | <0.001 | 15.937 | 40.574 | 0.852 | <0.001 |
| GH5(2) | IPR041036/PF18564 | 87 | 3.90 | 0.270 | <0.001 | 7.875 | 1.649 | 0.073 | <0.001 |
| GH6 | IPR016288/PF01341 | 289 | 1.17 | 0.915 | <0.001 | 11.217 | 41.034 | 0.837 | <0.001 |
| GH7 | IPR001722/PF00840 | 421 | 0.80 | NA | NA | NA | NA | NA | NA |
| GH8 | IPR002037/PF01270 | 326 | 1.04 | 0.823 | <0.001 | 17.583 | 37.007 | 0.677 | <0.001 |
| GH9 | IPR001701/PF00759 | 448 | 0.76 | 0.946 | <0.001 | 10.773 | 67.020 | 0.895 | <0.001 |
| GH10 | IPR001000/PF00331 | 319 | 1.06 | 0.941 | <0.001 | 11.416 | 44.305 | 0.885 | <0.001 |
| GH11 | IPR033123/PF00457 | 186 | 1.83 | 0.818 | <0.001 | 7.883 | 25.323 | 0.670 | <0.001 |
| GH12 | IPR002594/PF01670 | 208 | 1.63 | 0.680 | <0.001 | 16.400 | 20.378 | 0.463 | <0.001 |
| GH13 | IPR006047/PF00128 | 339 | 1.00 | 0.941 | <0.001 | 14.615 | 52.830 | 0.885 | <0.001 |
| GH14 | IPR001554/PF01373 | 422 | 0.80 | 0.701 | <0.001 | 21.000 | 40.167 | 0.492 | <0.001 |
| GH15 | IPR011613/PF00723 | 445 | 0.76 | 0.912 | <0.001 | 19.221 | 49.348 | 0.832 | <0.001 |
| GH16 | IPR000757/PF00722 | 180 | 1.88 | 0.851 | <0.001 | 9.315 | 26.201 | 0.724 | <0.001 |
| GH17 | IPR000490/PF00332 | 314 | 1.08 | 0.713 | <0.001 | 11.600 | 16.457 | 0.508 | <0.001 |
| GH18 | IPR001223/PF00704 | 357 | 0.95 | 0.928 | <0.001 | 11.041 | 47.604 | 0.861 | <0.001 |
| GH19 | IPR000726/PF00182 | 223 | 1.52 | 0.650 | <0.001 | 9.262 | 16.308 | 0.423 | <0.001 |
| GH20 | IPR015883/PF00728 | 346 | 0.98 | 0.869 | <0.001 | 15.935 | 45.303 | 0.755 | <0.001 |
| GH20(2) | IPR015882/PF02838 | 130 | 2.61 | 0.689 | <0.001 | 6.661 | 16.419 | 0.475 | <0.001 |
| GH20(3) | IPR041063/PF18088 | 183 | 1.85 | 0.285 | <0.001 | 20.728 | 2.504 | 0.081 | <0.001 |
| GH22 | IPR001916/PF00062 | 141 | 2.39 | 0.776 | 0.123 | 1.143 | 8.071 | 0.602 | 0.801 |
| GH23 | IPR008258/PF01464 | 106 | 3.21 | 0.815 | <0.001 | 13.739 | 18.359 | 0.665 | <0.001 |
| GH24 | IPR002196/PF00959 | 108 | 3.15 | 0.764 | <0.001 | 13.008 | 14.676 | 0.583 | <0.001 |
| GH25 | IPR002053/PF01183 | 177 | 1.91 | 0.915 | <0.001 | 6.625 | 25.179 | 0.837 | <0.001 |
| GH26 | IPR022790/PF02156 | 298 | 1.14 | 0.779 | <0.001 | 9.809 | 28.964 | 0.607 | <0.001 |
| GH27 | IPR002241/PF16499 | 286 | 1.18 | 0.930 | <0.001 | 12.346 | 41.717 | 0.865 | <0.001 |
| GH28 | IPR000743/PF00295 | 315 | 1.08 | 0.856 | <0.001 | 16.238 | 35.037 | 0.733 | <0.001 |
| GH29 | IPR000933/PF01120 | 332 | 1.02 | 0.896 | <0.001 | 12.482 | 47.102 | 0.803 | <0.001 |
| GH30 | IPR033453/PF02055 | 342 | 0.99 | 0.644 | <0.001 | 19.048 | 25.212 | 0.415 | <0.001 |
| GH30(2) | IPR033452/PF17189 | 68 | 4.95 | 0.624 | <0.001 | 5.247 | 5.649 | 0.390 | <0.001 |
| GH31 | IPR000322/PF01055 | 373 | 0.91 | 0.857 | <0.001 | 13.967 | 42.392 | 0.734 | <0.001 |
| GH31(2) | IPR025887/PF13802 | 204 | 1.66 | 0.738 | <0.001 | 12.243 | 17.477 | 0.545 | <0.001 |
| GH31(3) | IPR048395/PF21365 | 119 | 2.85 | 0.823 | <0.001 | 4.888 | 12.398 | 0.677 | <0.001 |
| GH32 | IPR013148/PF00251 | 320 | 1.06 | 0.918 | <0.001 | 5.284 | 50.113 | 0.843 | <0.001 |
| GH32(2) | IPR013189/PF08244 | 163 | 2.07 | 0.871 | <0.001 | -1.005 | 17.460 | 0.759 | 0.005 |
| GH33 | IPR011040/PF13088 | 288 | 1.18 | 0.802 | <0.001 | 0.837 | 54.430 | 0.644 | 0.505 |
| GH34 | IPR001860/PF00064 | 328 | 1.03 | NA | NA | NA | NA | NA | NA |
| GH35 | IPR031330/PF01301 | 324 | 1.04 | 0.746 | <0.001 | 28.049 | 30.984 | 0.556 | <0.001 |
| GH36 | NA/PF02065 | 338 | 1.00 | 0.817 | <0.001 | 13.566 | 36.779 | 0.668 | <0.001 |
| GH37 | IPR001661/PF01204 | 511 | 0.66 | 0.849 | <0.001 | 35.544 | 64.823 | 0.720 | <0.001 |
| GH38 | IPR000602/PF01074 | 275 | 1.23 | 0.863 | <0.001 | 13.333 | 35.422 | 0.746 | <0.001 |
| GH38(2) | IPR011682/PF07748 | 209 | 1.62 | 0.839 | <0.001 | 11.788 | 25.461 | 0.704 | <0.001 |
| GH38(3) | IPR015341/PF09261 | 96 | 3.54 | 0.839 | <0.001 | 6.207 | 12.119 | 0.704 | <0.001 |
| GH38(4) | IPR041147/PF17677 | 77 | 4.37 | 0.616 | <0.001 | 5.325 | 5.528 | 0.380 | <0.001 |
| GH38(6) | IPR041566/PF18230 | 88 | 3.85 | NA | NA | NA | NA | NA | NA |
| GH38(5) | IPR041509/PF18438 | 109 | 3.11 | 0.219 | <0.001 | 14.060 | 0.968 | 0.048 | <0.001 |
| GH39 | IPR049166/PF01229 | 462 | 0.73 | 0.442 | <0.001 | 26.607 | 13.620 | 0.195 | <0.001 |
| GH42 | IPR013529/PF02449 | 371 | 0.91 | 0.893 | <0.001 | 21.765 | 49.637 | 0.797 | <0.001 |
| GH42(2) | IPR013738/PF08532 | 202 | 1.68 | 0.883 | <0.001 | 8.451 | 27.389 | 0.780 | <0.001 |
| GH42(3) | IPR013739/PF08533 | 55 | 6.17 | 0.710 | <0.001 | 2.757 | 5.997 | 0.504 | <0.001 |
| GH43 | IPR006710/PF04616 | 294 | 1.15 | 0.953 | <0.001 | 12.252 | 45.162 | 0.908 | <0.001 |
| GH44 | IPR024745/PF12891 | 235 | 1.44 | 0.900 | <0.001 | 5.783 | 32.574 | 0.810 | <0.001 |
| GH45 | IPR000334/PF02015 | 202 | 1.67 | 0.992 | <0.001 | -9.872 | 40.255 | 0.984 | 0.013 |
| GH46 | IPR000400/PF01374 | 209 | 1.62 | 0.784 | <0.001 | 11.427 | 26.822 | 0.615 | <0.001 |
| GH47 | IPR001382/PF01532 | 477 | 0.71 | 0.923 | <0.001 | 4.211 | 57.047 | 0.852 | <0.001 |
| GH48 | IPR000556/PF02011 | 632 | 0.54 | 0.703 | <0.001 | 50.844 | 54.798 | 0.494 | <0.001 |
| GH49 | IPR005192/PF03718 | 116 | 2.92 | 0.146 | 0.652 | 18.000 | 4.273 | 0.021 | 0.068 |
| GH49(2) | IPR023226/PF17433 | 194 | 1.74 | -0.628 | 0.022 | 37.316 | -9.342 | 0.394 | <0.001 |
| GH50 | IPR040669/PF17992 | 194 | 1.74 | 0.593 | <0.001 | 10.791 | 15.459 | 0.352 | <0.001 |
| GH51 | IPR010720/PF06964 | 203 | 1.67 | 0.812 | <0.001 | 4.712 | 29.915 | 0.660 | <0.001 |
| GH52 | IPR000852/PF03512 | 418 | 0.81 | 0.485 | <0.001 | 38.096 | 26.661 | 0.235 | <0.001 |
| GH53 | IPR011683/PF07745 | 336 | 1.01 | 0.841 | <0.001 | 20.953 | 38.540 | 0.708 | <0.001 |
| GH54 | IPR015289/PF09206 | 321 | 1.06 | 0.936 | <0.001 | 10.837 | 48.060 | 0.877 | 0.004 |
| GH55 | IPR024535/PF12708 | 313 | 1.08 | 0.580 | <0.001 | 25.786 | 33.310 | 0.336 | <0.001 |
| GH56 | IPR018155/PF01630 | 335 | 1.01 | NA | NA | NA | NA | NA | NA |
| GH57 | IPR004300/PF03065 | 327 | 1.04 | 0.851 | <0.001 | 9.182 | 43.945 | 0.724 | <0.001 |
| GH58 | IPR030392/PF13884 | 57 | 5.97 | 0.052 | 0.107 | 12.882 | 5.203 | 0.003 | <0.001 |
| GH59 | IPR035394/PF17387 | 113 | 3.00 | 0.804 | <0.001 | 3.808 | 12.170 | 0.647 | 0.043 |
| GH62 | IPR005193/PF03664 | 271 | 1.25 | 0.901 | <0.001 | 11.670 | 39.339 | 0.811 | <0.001 |
| GH62(2) | IPR031335/PF03200 | 492 | 0.69 | -0.036 | 0.459 | 61.798 | -1.860 | 0.001 | <0.001 |
| GH63 | IPR031631/PF16923 | 219 | 1.55 | NA | NA | NA | NA | NA | NA |
| GH64 | IPR032477/PF16483 | 363 | 0.93 | 0.857 | <0.001 | 14.933 | 43.343 | 0.734 | <0.001 |
| GH65 | IPR005195/PF03632 | 370 | 0.92 | 0.891 | <0.001 | 10.673 | 50.763 | 0.793 | <0.001 |
| GH65(2) | IPR005194/PF03633 | 59 | 5.70 | 0.650 | <0.001 | 5.880 | 5.975 | 0.422 | <0.001 |
| GH65(3) | IPR005196/PF03636 | 248 | 1.36 | 0.837 | <0.001 | 10.222 | 29.938 | 0.700 | <0.001 |
| GH66 | IPR025092/PF13199 | 577 | 0.59 | 0.907 | <0.001 | 4.164 | 78.449 | 0.823 | 0.012 |
| GH67 | IPR011100/PF07488 | 317 | 1.07 | 0.532 | <0.001 | 27.093 | 23.087 | 0.283 | <0.001 |
| GH68 | IPR003469/PF02435 | 408 | 0.83 | 0.746 | <0.001 | 31.459 | 39.617 | 0.557 | <0.001 |
| GH70 | IPR003318/PF02324 | 847 | 0.40 | 0.913 | <0.001 | 18.260 | 132.140 | 0.834 | 0.144 |
| GH71 | IPR005197/PF03659 | 380 | 0.89 | 0.689 | <0.001 | 18.994 | 26.885 | 0.475 | <0.001 |
| GH72 | IPR004886/PF03198 | 308 | 1.10 | -0.469 | 0.037 | 23.824 | -18.157 | 0.220 | <0.001 |
| GH73 | IPR002901/PF01832 | 130 | 2.61 | 0.901 | <0.001 | 5.836 | 19.631 | 0.813 | <0.001 |
| GH75 | IPR009939/PF07335 | 160 | 2.11 | 0.773 | <0.001 | 8.016 | 17.490 | 0.597 | <0.001 |
| GH76 | IPR005198/PF03663 | 345 | 0.98 | 0.905 | <0.001 | 11.419 | 39.722 | 0.819 | <0.001 |
| GH77 | IPR003385/PF02446 | 492 | 0.69 | 0.711 | <0.001 | 30.243 | 50.533 | 0.506 | <0.001 |
| GH78 | IPR008902/PF05592 | 99 | 3.44 | 0.872 | <0.001 | 6.827 | 11.088 | 0.760 | <0.001 |
| GH78(2) | IPR013737/PF08531 | 166 | 2.04 | 0.867 | <0.001 | 8.125 | 17.618 | 0.752 | <0.001 |
| GH78(3) | IPR035396/PF17389 | 339 | 1.00 | 0.920 | <0.001 | 27.837 | 45.393 | 0.847 | <0.001 |
| GH78(4) | IPR035398/PF17390 | 71 | 4.77 | 0.826 | <0.001 | 3.456 | 7.177 | 0.682 | <0.001 |
| GH79 | IPR005199/PF03662 | 310 | 1.09 | 0.581 | <0.001 | 11.090 | 20.425 | 0.338 | <0.001 |
| GH79(2) | IPR031728/PF16862 | 107 | 3.17 | -0.328 | 0.472 | 10.600 | -2.800 | 0.108 | 0.083 |
| GH80 | IPR025880/PF13647 | 301 | 1.13 | NA | NA | NA | NA | NA | NA |
| GH81 | IPR040451/PF03639 | 319 | 1.06 | -0.028 | 0.654 | 23.136 | -0.624 | 0.001 | <0.001 |
| GH81(2) | IPR040720/PF17652 | 343 | 0.99 | 0.678 | <0.001 | 26.020 | 25.237 | 0.460 | <0.001 |
| GH83 | IPR000665/PF00423 | 539 | 0.63 | NA | NA | NA | NA | NA | NA |
| GH84 | IPR011496/PF07555 | 294 | 1.15 | 0.891 | <0.001 | 9.796 | 41.285 | 0.793 | <0.001 |
| GH85 | IPR005201/PF03644 | 301 | 1.12 | 0.546 | <0.001 | 30.257 | 21.761 | 0.298 | <0.001 |
| GH86 | IPR040527/PF18206 | 102 | 3.33 | 0.893 | <0.001 | 13.575 | 4.203 | 0.797 | 0.002 |
| GH88 | IPR010905/PF07470 | 332 | 1.02 | 0.667 | <0.001 | 45.485 | 71.938 | 0.445 | <0.001 |
| GH92 | IPR012939/PF07971 | 471 | 0.72 | 0.956 | <0.001 | 8.200 | 72.493 | 0.914 | <0.001 |
| GH92(2) | IPR041371/PF17678 | 238 | 1.43 | 0.934 | <0.001 | 2.631 | 35.459 | 0.872 | 0.005 |
| GH97 | IPR019563/PF10566 | 272 | 1.25 | 0.843 | <0.001 | 12.931 | 33.969 | 0.710 | <0.001 |
| GH97(2) | IPR029486/PF14508 | 257 | 1.32 | 0.801 | <0.001 | 11.933 | 27.904 | 0.642 | <0.001 |
| GH97(3) | IPR029483/PF14509 | 96 | 3.51 | 0.716 | <0.001 | 6.455 | 10.484 | 0.512 | <0.001 |
| GH98 | IPR013191/PF08306 | 313 | 1.08 | 0.080 | 0.330 | 46.484 | 2.944 | 0.006 | <0.001 |
| GH98(2) | IPR013190/PF08307 | 251 | 1.35 | -0.072 | 0.385 | 28.135 | -1.242 | 0.005 | <0.001 |
| GH99 | IPR026071/PF16317 | 324 | 1.04 | 0.750 | <0.001 | 10.020 | 30.402 | 0.563 | <0.001 |
| GH100 | IPR024746/PF12899 | 430 | 0.79 | 0.764 | <0.001 | 15.251 | 64.214 | 0.583 | <0.001 |
| GH101 | IPR035364/PF17451 | 116 | 2.91 | 0.252 | <0.001 | 16.323 | 3.939 | 0.063 | <0.001 |
| GH101(2) | IPR040633/PF18080 | 241 | 1.40 | 0.486 | <0.001 | 25.505 | 11.976 | 0.236 | <0.001 |
| GH106 | NA/PF17132 | 759 | 0.45 | 0.748 | <0.001 | 51.932 | 51.802 | 0.559 | <0.001 |
| GH108 | IPR008565/PF05838 | 85 | 3.99 | 0.755 | <0.001 | 4.143 | 12.133 | 0.570 | <0.001 |
| GH109 | IPR049303/PF21252 | 168 | 2.02 | 0.889 | <0.001 | 13.273 | 18.164 | 0.790 | <0.001 |
| GH110 | IPR039448/PF13229 | 220 | 1.54 | 0.101 | <0.001 | 56.220 | 29.398 | 0.010 | <0.001 |
| GH112 | IPR035080/PF09508 | 432 | 0.78 | 0.695 | <0.001 | 28.529 | 40.071 | 0.483 | <0.001 |
| GH112(2) | IPR035363/PF17385 | 219 | 1.55 | 0.671 | <0.001 | 11.815 | 23.875 | 0.450 | <0.001 |
| GH112(3) | IPR035356/PF17386 | 51 | 6.62 | 0.439 | <0.001 | 2.828 | 5.430 | 0.193 | <0.001 |
| GH114 | IPR004352/PF03537 | 226 | 1.50 | 0.534 | <0.001 | 30.017 | 16.504 | 0.285 | <0.001 |
| GH115 | IPR031924/PF15979 | 341 | 0.99 | 0.826 | <0.001 | 18.330 | 39.489 | 0.682 | <0.001 |
| GH115(2) | IPR041437/PF17829 | 175 | 1.94 | 0.523 | <0.001 | 17.732 | 11.962 | 0.273 | <0.001 |
| GH116 | IPR006775/PF04685 | 349 | 0.97 | 0.906 | <0.001 | 14.147 | 43.493 | 0.821 | <0.001 |
| GH116(2) | IPR024462/PF12215 | 326 | 1.04 | 0.894 | <0.001 | 10.329 | 31.511 | 0.800 | <0.001 |
| GH123 | IPR025150/PF13320 | 319 | 1.06 | 0.417 | <0.001 | 34.343 | 18.661 | 0.174 | <0.001 |
| GH123(2) | IPR045711/PF19543 | 944 | 0.36 | -0.331 | 0.001 | 57.386 | -15.778 | 0.109 | <0.001 |
| GH125 | IPR008313/PF06824 | 408 | 0.83 | 0.775 | <0.001 | 26.937 | 39.801 | 0.600 | <0.001 |
| GH127 | IPR012878/PF07944 | 384 | 0.88 | 0.658 | <0.001 | 30.061 | 69.137 | 0.434 | <0.001 |
| GH127(2) | IPR049049/PF20737 | 109 | 3.11 | 0.682 | <0.001 | 9.437 | 11.813 | 0.465 | <0.001 |
| GH129 | IPR021459/PF11308 | 317 | 1.07 | -0.100 | 0.009 | 51.027 | -14.027 | 0.010 | <0.001 |
| GH130 | IPR007184/PF04041 | 309 | 1.10 | 0.911 | <0.001 | 4.637 | 40.695 | 0.830 | <0.001 |
| GH131 | IPR041524/PF18271 | 245 | 1.38 | NA | NA | NA | NA | NA | NA |
